# Supplementary material for: Synaptic Dysfunction by Mutations in GRIN2B: Influence of Triheteromeric NMDA Receptors on Gain-of-Function and Loss-of-Function Mutant Classification
Source: Brain Sci. 2022 Jun 15;12(6):789. doi: 10.3390/brainsci12060789 (PMC9221112; doi:10.3390/brainsci12060789)
Supplement: Supplementary file 1 [file brainsci-12-00789-s001.zip › brainsci-1750947-supplementary.pdf]

Supplemental Information

Table of Contents

Figures S1.1 – S1.15: Summary of Linear Mixed Models (LMM)......2
Figure S1.1. NMDA-EPSC peak amplitudes in GluN2A/B double KO neurons rescued with GluN2B mutants......3
Figure S1.2. NMDA-EPSC charge transfer in GluN2A/B double KO neurons rescued with GluN2B mutants......4
Figure S1.3. NMDA-EPSC decay in GluN2A/B double KO neurons rescued with GluN2B GOF mutants......5
Figure S1.4. NMDA-EPSC peak amplitudes in GluN2B knockout neurons rescued with GluN2B mutants......6
Figure S1.5. AMPA-EPSC peak amplitudes in GluN2B knockout neurons rescued with GluN2B mutants......7
Figure S1.6. NMDA-EPSC decay in GluN2B knockout neurons rescued with GluN2B mutants......8
Figure S1.7. NMDA-EPSC charge transfer in GluN2B knockout neurons rescued with GluN2B mutants......9
Figure S1.8. NMDA-EPSC peak amplitude in GluN2B knockout neurons rescued with GluN2B mutants before and after application of TCN-201......10
Figure S1.9. NMDA-EPSC peak amplitude in GluN2A or 2B knockout neurons rescued with C436R mutants......11
Figure S1.10. NMDA-EPSC decay time constant in GluN2A or 2B knockout neurons rescued with C436R mutants......12
Figure S1.11. NMDA-EPSC charge transfer in GluN2A or 2B knockout neurons rescued with C436R mutants......13
Figure S1.12. NMDA-EPSC charge transfer in neurons expressing GluN2B knockout alleles......14
Figure S1.13. NMDA-EPSC peak amplitudes in neurons expressing GluN2B knockout alleles......15
Figure S1.14. NMDA-EPSC decay time constant in neurons expressing GluNB knockout alleles......16
Figure S1.15. NMDA-EPSC risetimes in neurons expressing GluN2B knockout alleles......17

***Figure S2: Effective rescue of native mouse GluN2B by human GluN2B*......18**

***Figure S3: GluN2B mutations are not associated with much effect on AMPAR-EPSCs.*......19**

***Figure S4: NMDA-EPSC decay is similar after selective inhibition of GluN2A-containing NMDA receptors*......20**

***Table S1. Matrix of orthogonal contrasts based on a priori clustering of mutations.*......21**

***Table S2: Primers used to generate mutant GluN2B constructs from WT pCI-Neo GRIN2B*......22**

***Table S3. Experiment sample sizes*......23**

***Table S4. Functional properties of NMDA receptor subtypes in HEK293T cells*......24**

***Table S5. Effects of TCN-201 on responses from NMDA receptors in HEK293T cells*......25**

Figures S1.1 – S1.15: Summary of Linear Mixed Models (LMM)

The top of each of the following figures (S1.1 – S1.15) includes: the `lmer` (Wilkinson-Rogers-Pinheiro-Bates) model formula, an ANOVA table with $F(df, df_{res})$ statistics, p-values, and Bayes factors for hypothesis tests of interests on fixed effects, and a table of group sample size and intraclass correlation (ICC) for nuisance random effects. The bottom of each of the following figures also includes a series of plots that can be used to evaluate the validity of LMM assumptions including homoscedasticity of model residuals and their likeness to a Normally distribution. **a**) Standardized residuals plotted against the fitted values with smoothed conditional mean (red) and conditional median accompanied by lower and upper quartiles (blue) ; **b**) Histogram of residual overlaid with fitted Gaussian distribution (red) and kernel density estimate (blue); **c**) quantile-quantile (Q-Q) plot with 95% confidence bands; and **d**) Stem-and-leaf plot of Cook's distances.

Figure S1.1. NMDA-EPSC peak amplitudes in GluN2A/B double KO neurons rescued with GluN2B mutants

Top. Summary tables based on a Linear Mixed Model (LMM) predicting NMDA-EPSC peak amplitude from the co-transfection of different *GRIN2B* mutants together with Cre-GFP in CA1 neurons of *Grin2a*$^{fl/fl}$*2b*$^{fl/fl}$ slices. Fixed effects are summarised as an ANOVA table with the interaction term followed up with Dunnett's step-down posthoc comparisons. The *p*-value and Bayes factor for the interaction term (mutation:transfection) indicate a highly significant effect of transfecting GluN2B mutants on the peak amplitude of NMDA-EPSCs in GluN2A/B double knockout (KO) neurons. Of the variance not accounted for by the fixed effects, 4%, 7%, 9% and 80% was explained by variability between recording pairs, slices, animals and by residual (unexplained) variance respectively. ***Bottom***. Model residuals appeared to be homoscedastic (**a**) and normally distributed (**b**, **c**), with very few outlying data points (**a, c**) an no influential data points (i.e. Cook's distance < 1.0) (**d**). A breakdown of samples sizes is reported in Table S3.

**Response**					
NMDA-EPSC peak amplitude (peak)					
**Formula**					
$\log(\boldsymbol{peak}) \sim mutation * transfection + (1 \mid animal/slice/pair)$					
**Source (Fixed)**	***F***	***df***	$\boldsymbol{df_{res}}$	***p***	$\boldsymbol{BF_{10}}$
mutation	4.26	4	8.9		
transfection	500.43	1	85		
mutation:transfection	17.04	4	85	<.001	4.2E+10
**Posthoc test**	***t***		***df***	$\boldsymbol{p_{adj}}$	
WT vs. None	7.27		85	<.001	
R540H vs. None	3.93		85	<.001	
R696H vs. None	3.10		85	.005	
C456Y vs. None	0.97		85	.34	
**Source (random)**	***N***	**ICC**			
pair:(slice:animal)	90	.04			
slice:animal	57	.07			
animal	14	.09			
Residual		.80			

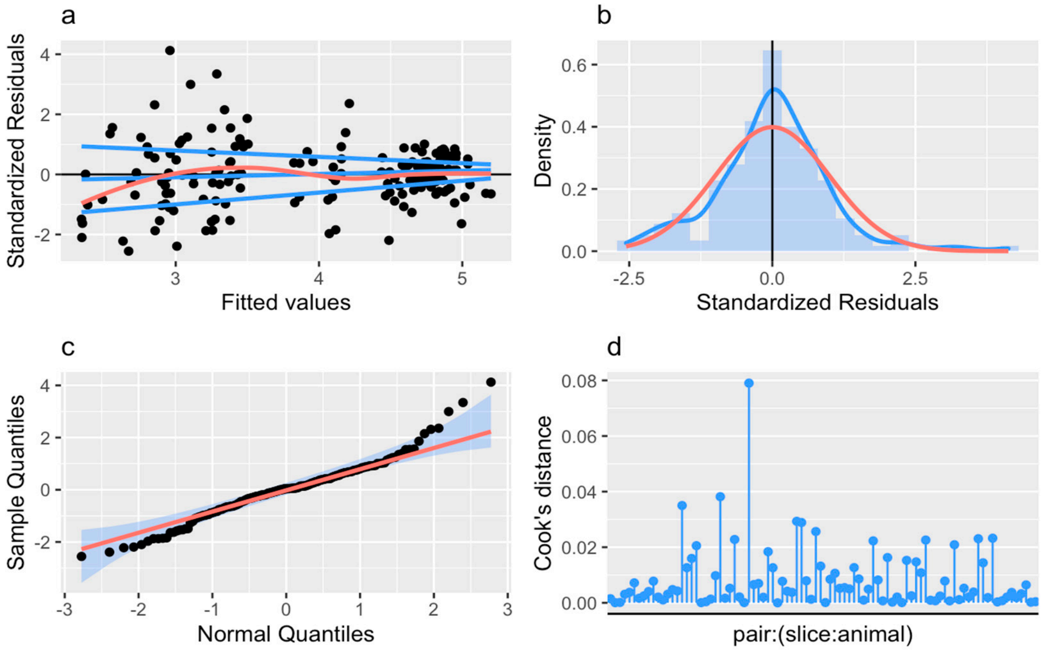

Figure S1.2. NMDA-EPSC charge transfer in GluN2A/B double KO neurons rescued with GluN2B mutants

Top. Summary tables based on a Linear Mixed Model (LMM) predicting NMDA-EPSC charge transfer from the co-transfection of different *GRIN2B* mutants together with Cre-GFP in CA1 neurons of *Grin2a$^{fl/fl}$2b$^{fl/fl}$* slices. Fixed effects are summarised as an ANOVA table with the interaction term followed up with Dunnett's step-down posthoc comparisons. The *p*-value and Bayes factor for the interaction term (mutation:transfection) indicate a highly significant effect of transfecting GluN2B mutants on the peak amplitude of NMDA-EPSCs in GluN2A/B double knockout (KO) neurons. Of the variance not accounted for by the fixed effects, 7%, 0%, 2% and 91% was explained by variability between recording pairs, slices, animals and by residual (unexplained) variance respectively. ***Bottom***. Model residuals appeared to be homoscedastic (**a**), normally distributed (**b**, **c**), without overt outliers (**a, c**) and without influential data points (i.e. Cook's distance < 1.0) (**d**). A breakdown of samples sizes is reported in Table S3.

**Response**					
NMDA-EPSC charge transfer					
**Formula**					
$\log(\boldsymbol{charge}) \sim mutation * transfection + (1\|animal/slice/pair)$					
**Source (Fixed)**	***F***	***df***	df_{res}	***p***	BF_{10}
mutation	10.62	3	6.87		
transfection	41.57	1	67		
mutation:transfection	23.65	3	67	<.001	4.3E+11
**Posthoc test**	***t***		***df***	p_{adj}	
R540H vs. WT	-1.01		67	.32	
R696H vs. WT	-1.45		67	.27	
C456Y vs. WT	-7.62		67	<.001	
**Source (random)**	***N***	**ICC**			
pair:(slice:animal)	71	.07			
slice:animal	46	.00			
animal	11	.02			
Residual		.91			

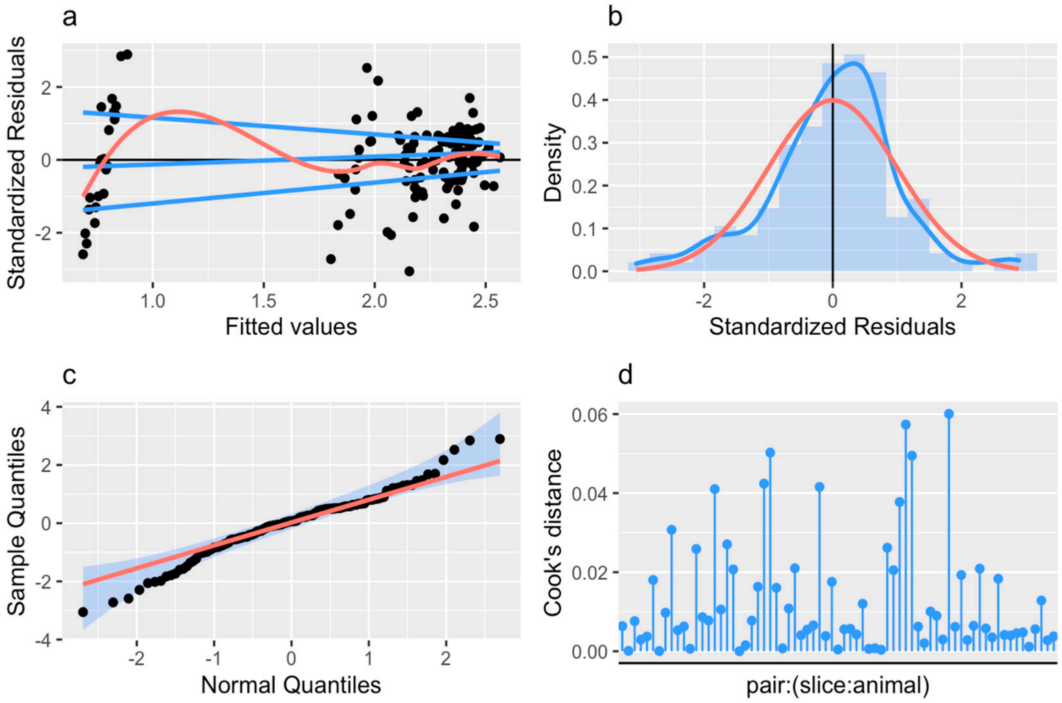

Figure S1.3. NMDA-EPSC decay in GluN2A/B double KO neurons rescued with GluN2B GOF mutants

Top. Summary tables based on a Linear Mixed Model (LMM) predicting NMDA-EPSC decay time constant from the co-transfection of different *GRIN2B* GoF mutants together with Cre-GFP in CA1 neurons of *Grin2a*$^{fl/fl}$*2b*$^{fl/fl}$ slices. Fixed effects are summarised as an ANOVA table with the interaction term followed up with Dunnett's step-down posthoc comparisons. The *p*-value and Bayes factor for the interaction term (mutation:transfection) indicate a significant effect of transfecting GluN2B GoF mutants on the decay time constant of NMDA-EPSCs in GluN2A/B double knockout (KO) neurons. All of the variance not accounted for by the fixed effects was residual, unexplained variance. ***Bottom***. Model residuals appeared to be homoscedastic (**a**), normally distributed (**b**, **c**), without overt outliers (**a, c**) and without influential data points (i.e. Cook's distance < 1.0) (**d**). A breakdown of samples sizes is reported in Table S3.

**Response**					
NMDA-EPSC decay time constant (decay)					
**Formula**					
$\log(\boldsymbol{decay}) \sim mutation * transfection + (1 \mid animal/slice/pair)$					
**Source (Fixed)**	***F***	***df***	***df***$_{res}$	***p***	***BF***$_{10}$
mutation	8.44	2	4.56		
transfection	244.54	1	44		
mutation:transfection	3.81	2	44	.03	6.19
**Posthoc test**	***t***		***df***	***p***$_{adj}$	
R540H vs. WT	2.32		44	.046	
R696H vs. WT	2.33		44	.046	
**Source (random)**	***N***	**ICC**			
pair:(slice:animal)	47	0			
slice:animal	32	0			
animal	8	0			
Residual		1			

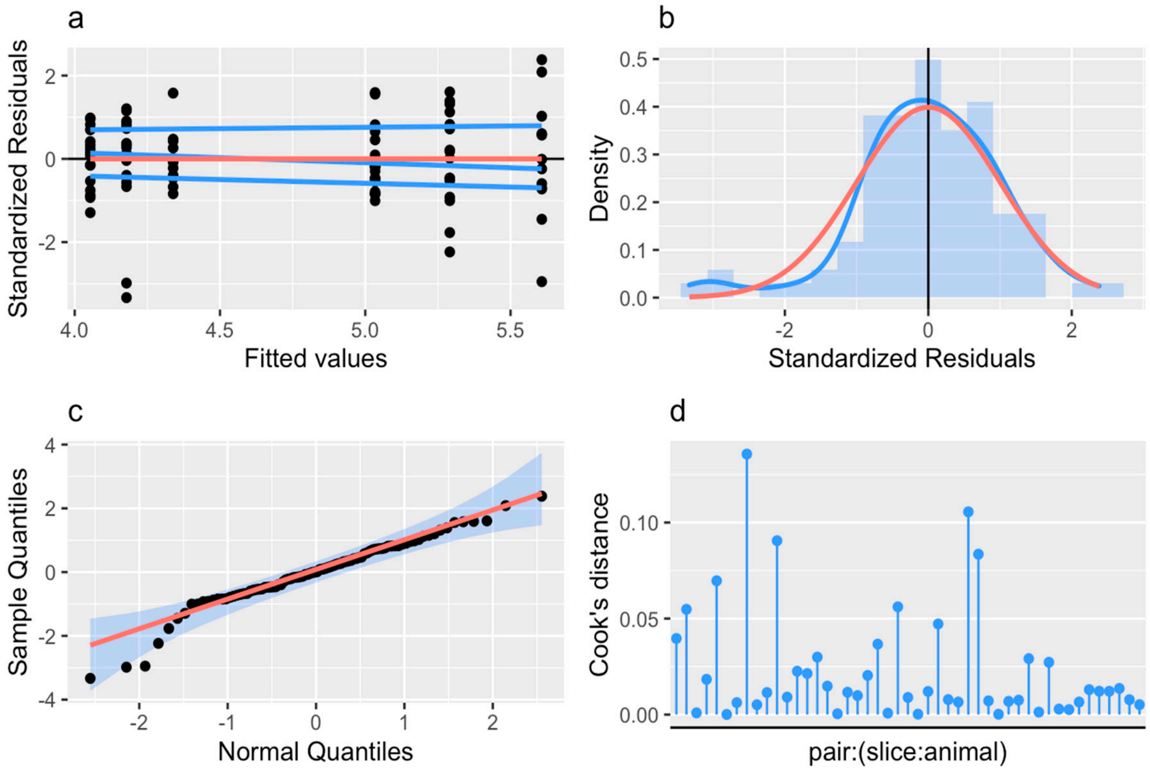

Figure S1.4. NMDA-EPSC peak amplitudes in GluN2B knockout neurons rescued with GluN2B mutants.

Top. Summary tables based on a Linear Mixed Model (LMM) predicting NMDA-EPSC peak amplitude from the co-transfection of different *GRIN2B* GoF mutants together with Cre-GFP in CA1 neurons of *Grin2b*$^{fl/fl}$ slices. Fixed effects are summarised as an ANOVA table with the interaction term decomposed into orthogonal contrasts (A-D, see Table S1). The Bayes factor for the interaction term (mutation:transfection) provides evidence more in favour of no effect of GluN2B mutants on the peak amplitude of NMDA-EPSCs in GluN2B knockout (KO) neurons. Of the variance not accounted for by the fixed effects, 16%, 27%, 36% and 21% was explained by variability between recording pairs, slices, animals, and residual (unexplained) variance respectively. ***Bottom***. Model residuals appeared to be homoscedastic (**a**), normally distributed (**b**, **c**), without overt outliers (**a, c**) and without influential data points (i.e. Cook's distance < 1.0) (**d**). A breakdown of samples sizes is reported in Table S3.

**Response**					
NMDA-EPSC peak amplitude (peak)					
**Formula**					
$\log(\boldsymbol{peak}) \sim mutation * transfection + (1 \mid animal/slice/pair)$					
**Source (Fixed)**	***F***	***df***	***df$_{res}$***	***p***	***BF$_{10}$***
mutation	2.91	4	16.22		
transfection	49.75	1	90		
mutation:transfection	0.21	4	90	.93	0.052
A. WT vs R540H, R696H, C456Y, C461F	0.38	1	90	.54	
B. C456Y, C461F vs R540H, R696H	0.40	1	90	.53	
C. C456Y vs. C461F	0.07	1	90	.80	
D. R540H vs. R696H	0.04	1	90	.85	
**Source (random)**	***N***	**ICC**			
pair:(slice:animal)	95	.16			
slice:animal	62	.27			
animal	22	.36			
Residual		.21			

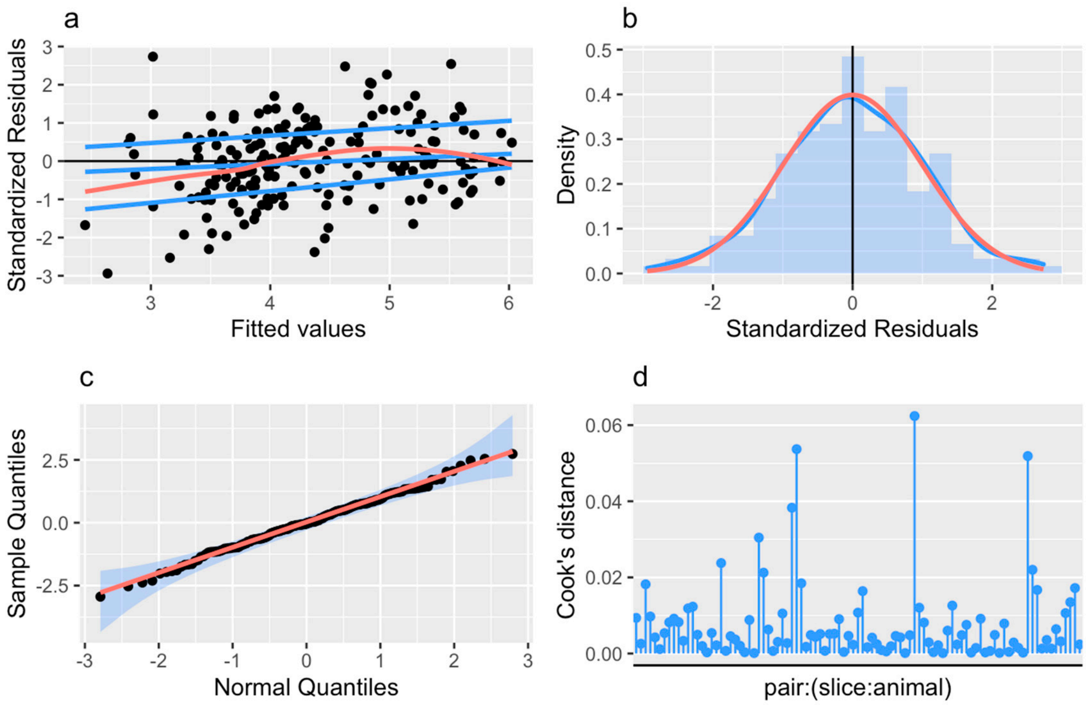

Figure S1.5. AMPA-EPSC peak amplitudes in GluN2B knockout neurons rescued with GluN2B mutants.

Top. Summary tables based on a Linear Mixed Model (LMM) predicting AMPA-EPSC peak amplitude from the co-transfection of different *GRIN2B* mutants together with Cre-GFP in CA1 neurons of *Grin2b*$^{fl/fl}$ slices. Fixed effects are summarised as an ANOVA table with the interaction term decomposed into orthogonal contrasts (A-D, see Table S1). The Bayes factor for the interaction term (mutation:transfection) provides reasonable evidence for no effect of GluN2B mutants on the peak amplitude of AMPA-EPSCs in GluN2B knockout (KO) neurons. Of the variance not accounted for by the fixed effects, 9%, 18%, 34% and 39% was explained by variability between recording pairs, slices, animals, and residual (unexplained) variance respectively. ***Bottom***. Model residuals appeared to be homoscedastic (**a**), normally distributed (**b**, **c**), without overt outliers (**a, c**) and without influential data points (i.e. Cook's distance < 1.0) (**d**). A breakdown of samples sizes is reported in Table S3.

**Response**					
AMPA-EPSC peak amplitude					
**Formula**					
$\log(\boldsymbol{peak}) \sim mutation * transfection + (1 \vert animal/slice/pair)$					
**Source (Fixed)**	***F***	***df***	***df$_{res}$***	***p***	***BF$_{10}$***
mutation	9.08	4	16.25		
transfection	0.77	1	90		
mutation:transfection	1.17	4	90	.33	0.197
A. WT vs R540H, R696H, C456Y, C461F	0.18	1	90	.68	
B. C456Y, C461F vs R540H, R696H	1.22	1	90	.27	
C. C456Y vs. C461F	3.41	1	90	.069	
D. R540H vs. R696H	0.14	1	90	.71	
**Source (random)**	***N***	**ICC**			
pair:(slice:animal)	95	.09			
slice:animal	62	.18			
animal	22	.34			
Residual		.39			

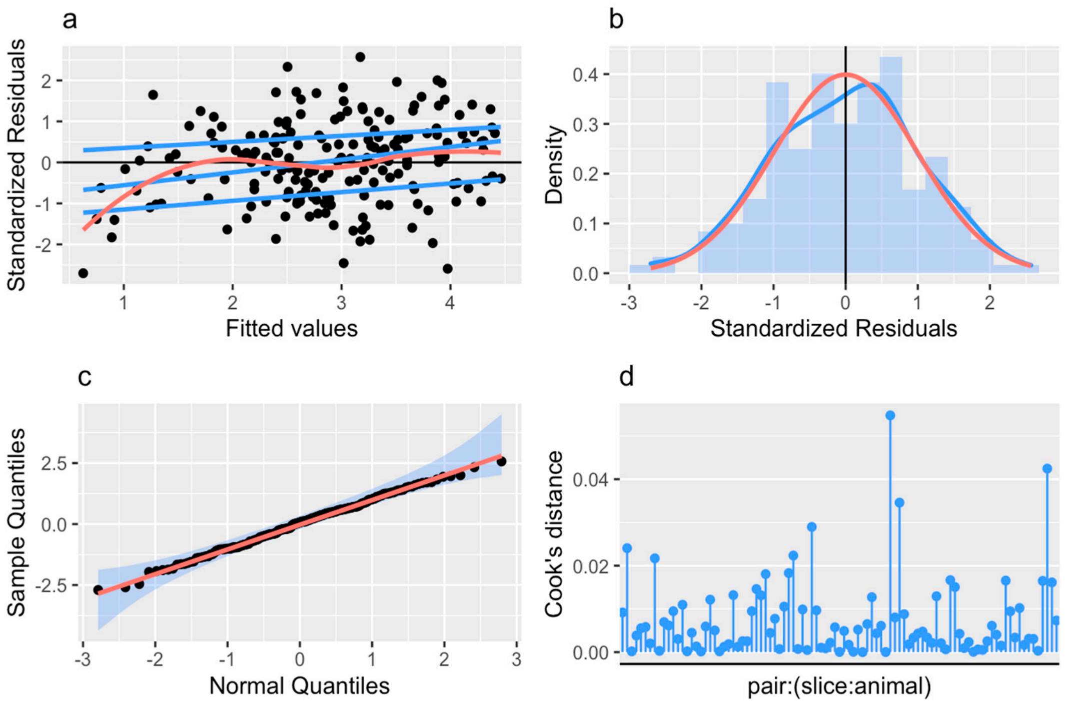

Figure S1.6. NMDA-EPSC decay in GluN2B knockout neurons rescued with GluN2B mutants.

Top. Summary tables based on a Linear Mixed Model (LMM) predicting NMDA-EPSC decay time constant from the co-transfection of different *GRIN2B* mutants together with Cre-GFP in CA1 neurons of *Grin2b*$^{fl/fl}$ slices. Fixed effects are summarised as an ANOVA table with the interaction term decomposed into orthogonal contrasts (A-D, see Table S1). The *p*-value and Bayes factor for the interaction term (mutation:transfection) indicate a highly significant effect of transfecting GluN2B mutants on the decay time constant of NMDA-EPSCs in GluN2B knockout (KO) neurons. Of the variance not accounted for by the fixed effects, 0%, 0%, 19% and 81% was explained by variability between recording pairs, slices, animals, and residual (unexplained) variance respectively. ***Bottom***. Model residuals appeared to be homoscedastic (**a**), normally distributed (**b**, **c**), without overt outliers (**a, c**) and without influential data points (i.e. Cook's distance < 1.0) (**d**). A breakdown of samples sizes is reported in Table S3.

**Response**					
NMDA-EPSC decay time constant (decay)					
**Formula**					
$\log(\boldsymbol{decay}) \sim mutation * transfection + (1 \mid animal/slice/pair)$					
**Source (Fixed)**	***F***	***df***	***df$_{res}$***	***p***	***BF$_{10}$***
mutation	2.67	4	15.78		
transfection	48.45	1	90		
mutation:transfection	8.85	4	90	<.001	1.52E+05
A. WT vs R540H, R696H, C456Y, C461F	19.20	1	90	<.001	
B. C456Y, C461F vs R540H, R696H	17.17	1	90	<.001	
C. C456Y vs. C461F	0.76	1	90	.39	
D. R540H vs. R696H	0.24	1	90	.63	
**Source (random)**	***N***	**ICC**			
pair:(slice:animal)	95	.00			
slice:animal	62	.00			
animal	22	.19			
Residual		.81			

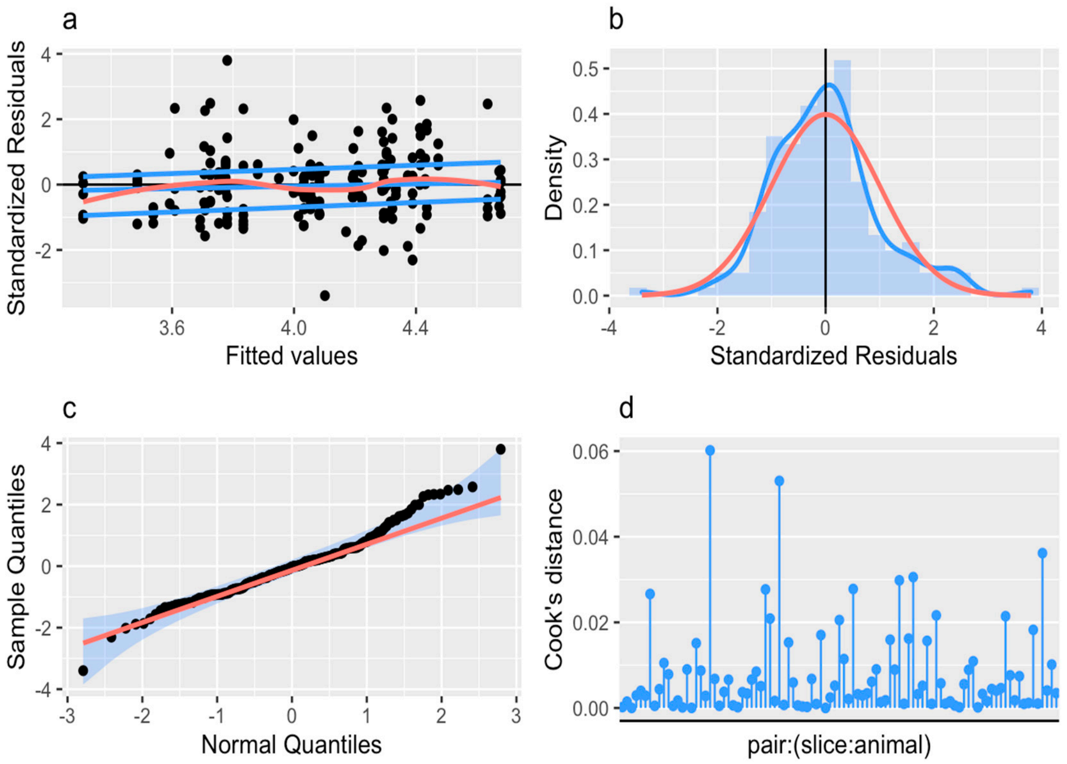

Figure S1.7. NMDA-EPSC charge transfer in GluN2B knockout neurons rescued with GluN2B mutants.

Top. Summary tables based on a Linear Mixed Model (LMM) predicting NMDA-EPSC charge transfer from the co-transfection of different *GRIN2B* mutants together with Cre-GFP in CA1 neurons of *Grin2b*$^{fl/fl}$ slices. Fixed effects are summarised as an ANOVA table with the interaction term decomposed into orthogonal contrasts (A-D, see Table S1). The p-value and Bayes factor for the interaction term (mutation:transfection) indicate a significant effect of GluN2B mutants on the charge transfer of NMDA-EPSCs in GluN2B knockout (KO) neurons. Of the variance not accounted for by the fixed effects, 9%, 22%, 36% and 33% was explained by variability between recording pairs, slices, animals, and residual (unexplained) variance respectively. ***Bottom***. Model residuals appeared to be homoscedastic (**a**), normally distributed (**b**, **c**), without overt outliers (**a, c**) and without influential data points (i.e. Cook's distance < 1.0) (**d**). A breakdown of samples sizes is reported in Table S3.

**Response**					
NMDA-EPSC charge transfer (charge)					
**Formula**					
$\log(\boldsymbol{charge}) \sim mutation * transfection + (1 \mid animal/slice/pair)$					
**Source (Fixed)**	***F***	***df***	***df$_{res}$***	***p***	***BF$_{10}$***
mutation	3.00	4	16.28		
transfection	57.13	1	90		
mutation:transfection	3.63	4	90	.009	5.16
A. WT vs R540H, R696H, C456Y, C461F	4.81	1	90	.031	
B. C456Y, C461F vs R540H, R696H	10.02	1	90	.002	
C. C456Y vs. C461F	0.75	1	90	.39	
D. R540H vs. R696H	0.13	1	90	.72	
**Source (random)**	***N***	**ICC**			
pair:(slice:animal)	95	.09			
slice:animal	62	.22			
animal	22	.36			
Residual		.33			

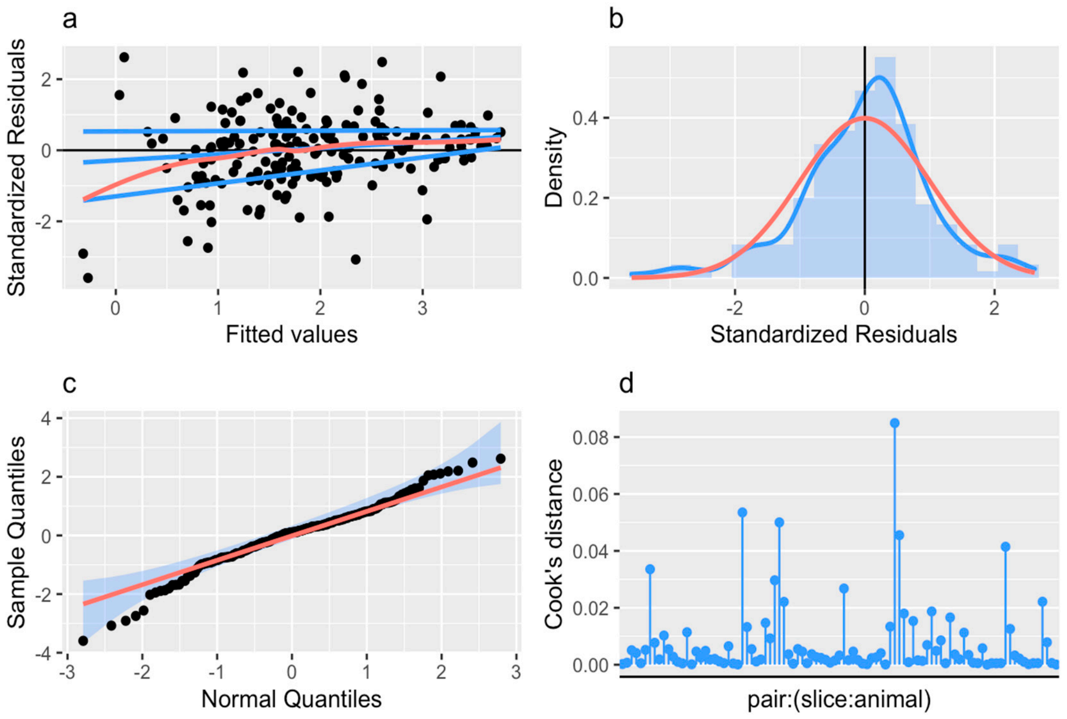

Figure S1.8. NMDA-EPSC peak amplitude in GluN2B knockout neurons rescued with GluN2B mutants before and after application of TCN-201.

Top. Summary tables based on a Linear Mixed Model (LMM) predicting the effect of TCN-201 on NMDA-EPSC peak amplitude from the co-transfection of different *GRIN2B* mutants together with Cre-GFP in CA1 neurons of *Grin2b*$^{fl/fl}$ slices. Fixed effects are summarised as an ANOVA table with the interaction term decomposed into orthogonal contrasts and followed up with pairwise comparisons by Westfall stepwise posthoc tests. The *p*-value and Bayes factor for the interaction term (mutation:drug) indicate a significant effect of TCN-201 on NMDA-EPSC peak amplitude depending on the GluN2B mutant expressed in GluN2B knockout (KO) neurons. Of the variance not accounted for by the fixed effects, 47, 2, 9 and 44% was explained by variability between cells, slices, animals and residual (unexplained) variance respectively. ***Bottom***. Model residuals appeared to be homoscedastic (**a**), normally distributed (**b**, **c**), without overt outliers (**a, c**) and without influential data points (i.e. Cook's distance < 1.0) (**d**). A breakdown of samples sizes is reported in Table S3.

**Response**					
NMDA-EPSC peak amplitude (peak)					
**Formula**					
$\log(\boldsymbol{peak}) \sim mutation * drug + (1\|animal/slice/cell)$					
**Source (Fixed)**	***F***	***df***	***df$_{res}$***	***p***	***BF$_{10}$***
mutation	2.79	2	1.71		
drug	122.3	1	35		
mutation:drug	3.95	2	35	.028	2.83
A. WT vs R696H, C456Y	7.81	1	35	.008	
B. R696H vs C456Y	0.03	1	35	.86	
**Posthoc test**	***t***		***df***	***p$_{adj}$***	
R696H vs. WT	-2.37		35	.035	
C456Y vs. WT	-2.61		35	.035	
C456Y vs. R696H	-0.18		35	.86	
**Source (random)**	***N***	**ICC**			
cell	38	.47			
slice	26	.02			
animal	5	.09			
Residual		.42			

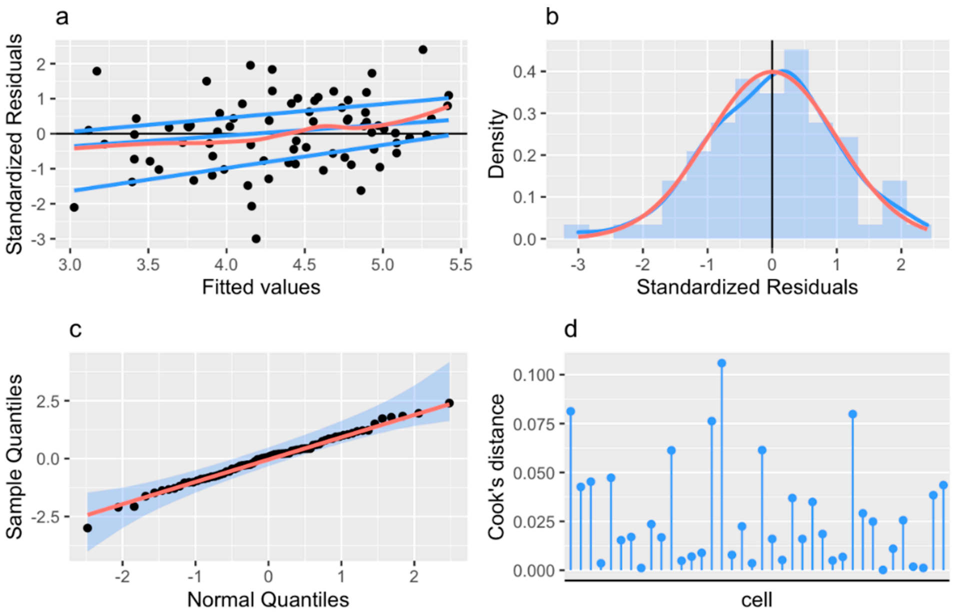

Figure S1.9. NMDA-EPSC peak amplitude in GluN2A or 2B knockout neurons rescued with C436R mutants.

Top. Summary tables based on a LMM predicting NMDA-EPSC peak amplitude from the co-transfection of the C436R mutant in *GRIN2A* or *GRIN2B* subunits together with Cre-GFP in CA1 neurons of *Grin2a*$^{fl/fl}$ *or Grin2b*$^{fl/fl}$ slices respectively. Fixed effects are summarised as an ANOVA table with the interaction term decomposed into orthogonal contrasts (A-D see Table S1). The Bayes factor for the interaction term (subunit:transfection) provides reasonable evidence for no effect of the subunit (GluN2A or 2B) on the peak amplitude of NMDA-EPSCs in KO neurons rescued with the C436R mutants. Of the variance not accounted for by the fixed effects, 40%, 40%, 0% and 20% was explained by variability between recording pairs, slices, animals, and residual (unexplained) variance respectively. ***Bottom***. Model residuals appeared to be homoscedastic (**a**), normally distributed (**b**, **c**), without overt outliers (**a, c**) and without influential data points (i.e. Cook's distance < 1.0) (**d**). A breakdown of samples sizes is reported in Table S3.

**Response**					
NMDA-EPSC peak amplitude (peak)					
**Formula**					
$\log(\boldsymbol{peak}) \sim subunit * transfection + (1 \mid animal/slice/pair)$					
**Source (Fixed)**	***F***	***df***	df_{res}	***p***	BF_{10}
subunit	18.19	1	5.27		
transfection	38.33	1	31		
subunit:transfection	0.18	1	31	.67	0.368
**Source (random)**	***N***	**ICC**			
pair:(slice:animal)	33	.40			
slice:animal	21	.40			
animal	9	.00			
Residual		.20			

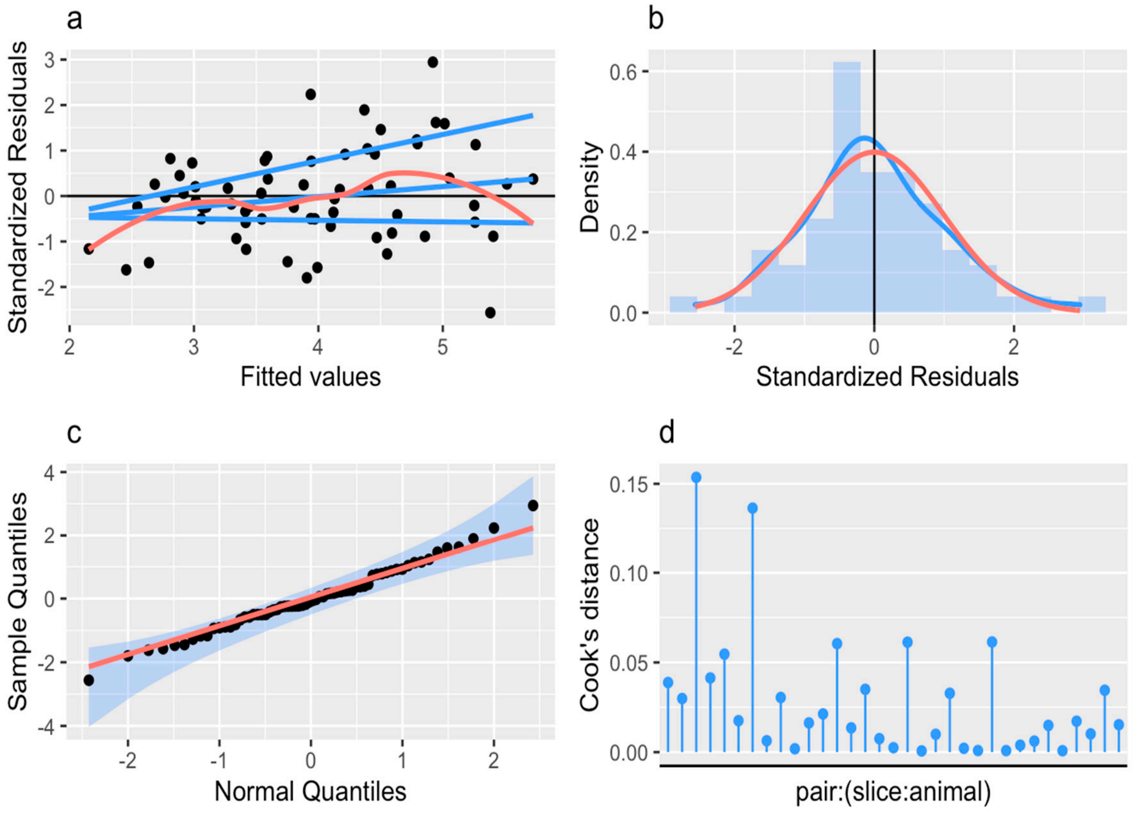

Figure S1.10. NMDA-EPSC decay time constant in GluN2A or 2B knockout neurons rescued with C436R mutants.

Top. Summary tables based on a LMM predicting NMDA-EPSC decay time constant from the co-transfection of the C436R mutant in *GRIN2A* or *GRIN2B* subunits together with Cre-GFP in CA1 neurons of *Grin2a*$^{fl/fl}$ *or Grin2b*$^{fl/fl}$ slices respectively. Fixed effects are summarised as an ANOVA table with the interaction term decomposed into orthogonal contrasts (A-D see Table S1). The *p*-value and Bayes factor for the interaction term (subunit:transfection) indicate a highly significant effect of transfecting different subunits (GluN2A or 2B) of the C436R mutant on the decay time constant of NMDA-EPSCs in KO neurons. Of the variance not accounted for by the fixed effects, 0%, 0%, 17% and 83% was explained by variability between recording pairs, slices, animals, and residual (unexplained) variance respectively. ***Bottom***. Model residuals appeared to be homoscedastic (**a**), normally distributed (**b**, **c**), without overt outliers (**a, c**) and without influential data points (i.e. Cook's distance < 1.0) (**d**). A breakdown of samples sizes is reported in Table S3.

**Response**					
NMDA-EPSC decay time constant (decay)					
**Formula**					
$\log(\boldsymbol{decay}) \sim subunit * transfection + (1\|animal/slice/pair)$					
**Source (Fixed)**	***F***	***df***	***df$_{res}$***	***p***	***BF$_{10}$***
subunit	41.16	1	5.73		
transfection	0.02	1	31		
subunit:transfection	40.71	1	31	<.001	5.47E+6
**Source (random)**	***N***	**ICC**			
pair:(slice:animal)	33	.00			
slice:animal	21	.00			
animal	9	.17			
Residual		.83			

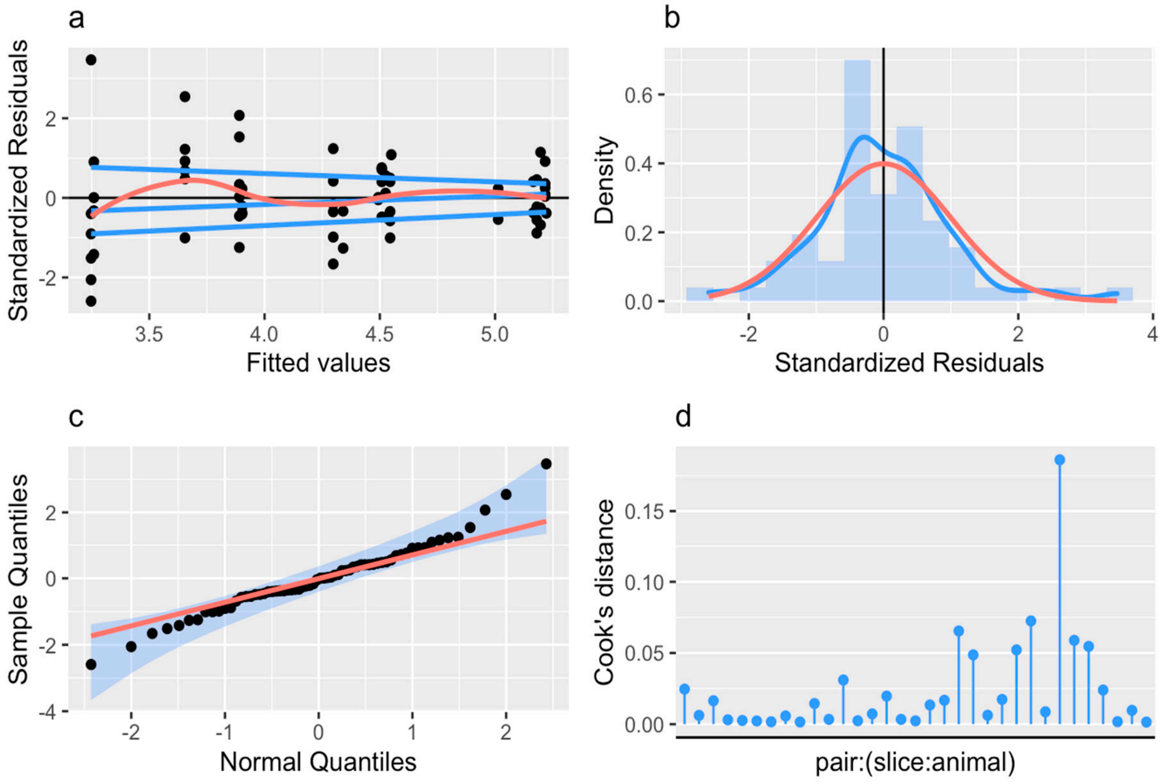

Figure S1.11. NMDA-EPSC charge transfer in GluN2A or 2B knockout neurons rescued with C436R mutants.

Top. Summary tables based on a LMM predicting NMDA-EPSC charge transfer from the co-transfection of the C436R mutant in *GRIN2A* or *GRIN2B* subunits together with Cre-GFP in CA1 neurons of *Grin2a*$^{fl/fl}$ *or Grin2b*$^{fl/fl}$ slices respectively. Fixed effects are summarised as an ANOVA table with the interaction term decomposed into orthogonal contrasts (A-D see Table S1). The *p*-value and Bayes factor for the interaction term (subunit:transfection) indicate a highly significant effect of transfecting different subunits (GluN2A or 2B) of the C436R mutant on the charge transfer of NMDA-EPSCs in KO neurons. Of the variance not accounted for by the fixed effects, 11%, 53%, 0% and 36% was explained by variability between recording pairs, slices, animals, and residual (unexplained) variance respectively. ***Bottom***. Model residuals appeared to be homoscedastic (**a**), normally distributed (**b**, **c**), without overt outliers (**a, c**) and without influential data points (i.e. Cook's distance < 1.0) (**d**). A breakdown of samples sizes is reported in Table S3.

**Response**					
NMDA-EPSC charge transfer					
**Formula**					
$\log(\boldsymbol{charge}) \sim subunit * transfection + (1\|animal/slice/pair)$					
**Source (Fixed)**	***F***	***df***	***df_{res}***	***p***	***BF₁₀***
subunit	32.11	1	5.32		
transfection	10.16	1	31		
subunit:transfection	19.73	1	31	<.001	468
**Source (random)**	***N***	**ICC**			
pair:(slice:animal)	96	.11			
slice:animal	62	.53			
animal	22	.00			
Residual		.36			

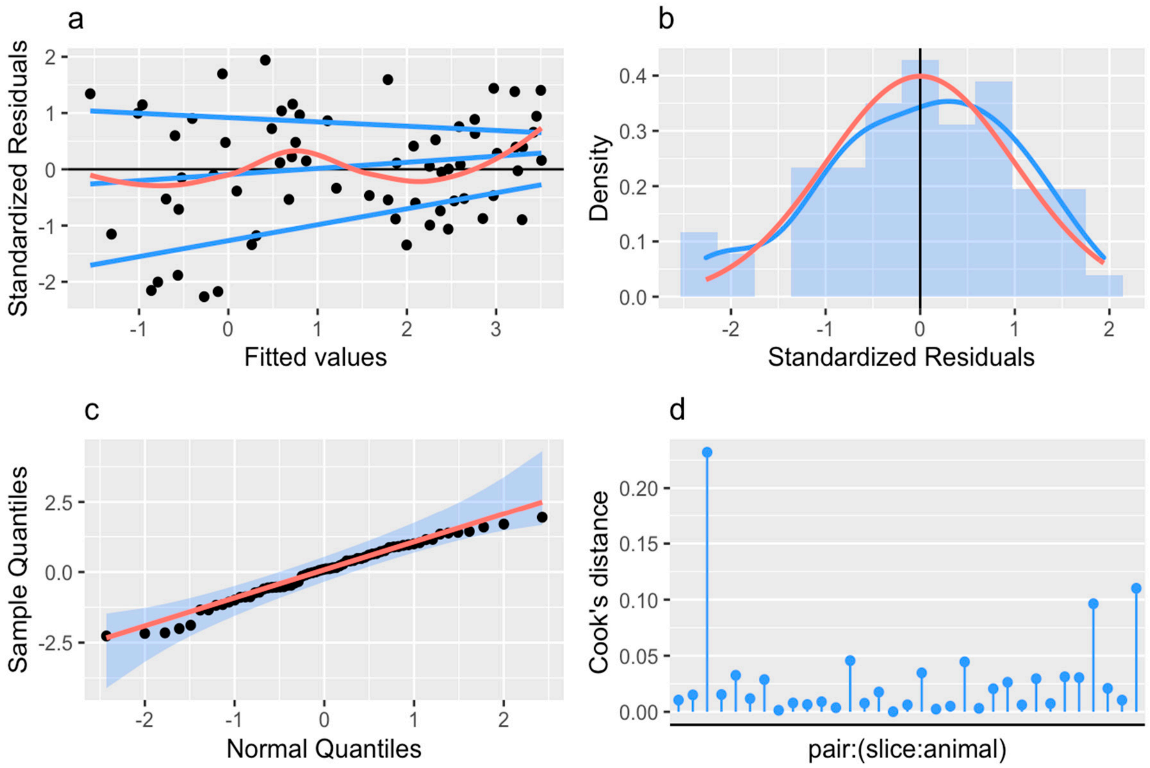

Figure S1.12. NMDA-EPSC charge transfer in neurons expressing GluN2B knockout alleles.

Top. Summary tables based on a LMM predicting NMDA-EPSC charge transfer from the transfection of Cre-GFP in CA1 neurons of slices with different genotypes of the floxed *Grin2b* allele. Fixed effects are summarised as an ANOVA table with the interaction term split into polynomial contrasts and followed up with pairwise comparisons by Westfall stepwise posthoc tests. The *p*-value and Bayes factor for the interaction term (subunit:transfection) indicate a highly significant effect of loss-of-function (null) GluN2B alleles on the charge transfer of NMDA-EPSCs. Of the variance not accounted for by the fixed effects, 15%, 35%, 10% and 40% was explained by variability between recording pairs, slices, animals, and residual (unexplained) variance respectively. ***Bottom***. Model residuals appeared to be homoscedastic (**a**), normally distributed (**b**, **c**), without overt outliers (**a, c**) and without influential data points (i.e. Cook's distance < 1.0) (**d**). A breakdown of samples sizes is reported in Table S3.

**Response**					
NMDA-EPSC charge transfer (charge)					
**Formula**					
$\log(\boldsymbol{charge}) \sim genotype * transfection + (1 \vert animal/slice/pair)$					
**Source (Fixed)**	***F***	***df***	***df_{res}***	***p***	***BF_{10}***
genotype	5.15	2	13.6		
transfection	106.45	1	104		
genotype:transfection	61.7	2	104	<.001	1.6E+17
Linear	94.0	1	104	<.001	
Quadratic	25.9	1	104	<.001	
**Posthoc test**	***t***		***df***	***p_{adj}***	
+/+ vs. +/-	-0.53		104	.60	
+/+ vs. -/-	-9.70		104	<.001	
+/- vs. -/-	-9.45		104	<.001	
**Source (random)**	***N***	***ICC***			
pair:(slice:animal)	107	.15			
slice:animal	56	.35			
animal	22	.10			
Residual		.40			

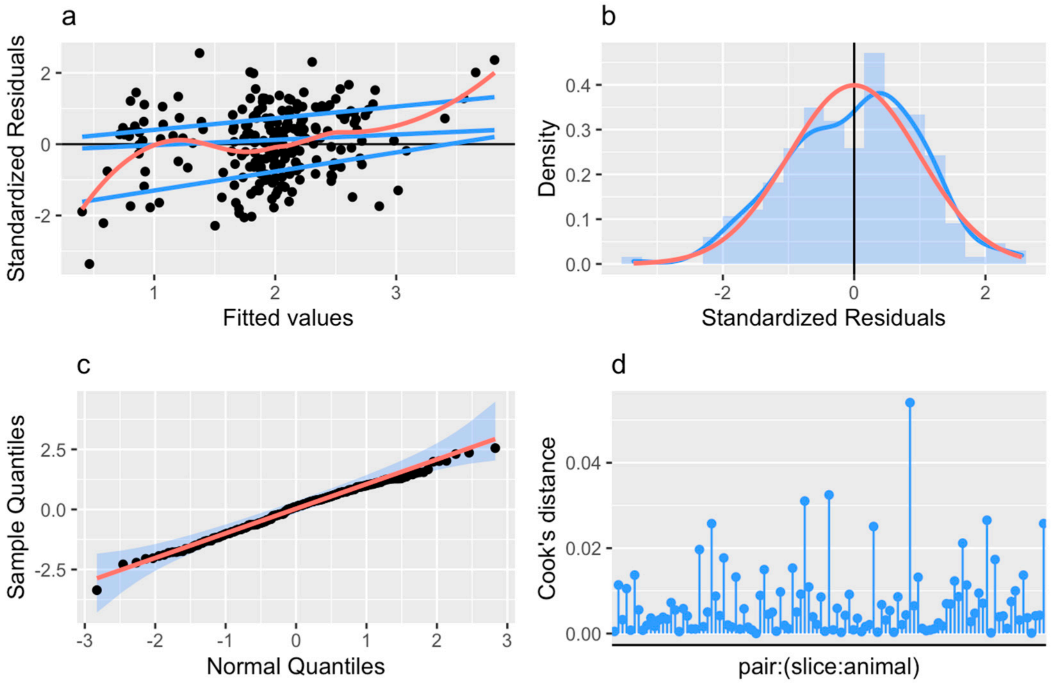

Figure S1.13. NMDA-EPSC peak amplitudes in neurons expressing GluN2B knockout alleles.

Top. Summary tables based on a LMM predicting NMDA-EPSC peak amplitudes from the transfection of Cre-GFP in CA1 neurons of slices with different genotypes of the floxed *Grin2b* allele. Fixed effects are summarised as an ANOVA table with the interaction term split into polynomial contrasts and followed up with pairwise comparisons by Westfall stepwise posthoc tests. The *p*-value and Bayes factor for the interaction term (subunit:transfection) indicate a highly significant effect of loss-of-function (null) GluN2B alleles on the charge transfer of NMDA-EPSCs. Of the variance not accounted for by the fixed effects, 14%, 33%, 6% and 47% was explained by variability between slices, animals, and residual (unexplained) variance respectively. ***Bottom***. Model residuals appeared to be homoscedastic (**a**), normally distributed (**b**, **c**), without overt outliers (**a, c**) and without influential data points (i.e. Cook's distance < 1.0) (**d**). A breakdown of samples sizes is reported in Table S3.

**Response**					
NMDA-EPSC peak amplitude (peak)					
**Formula**					
$\log(\boldsymbol{peak}) \sim genotype * transfection + (1 \mid animal/slice/pair)$					
**Source (Fixed)**	***F***	***df***	df_{res}	***p***	BF_{10}
genotype	1.53	2	13.08		
transfection	36.50	1	104		
genotype:transfection	17.57	2	104	<.001	2.04E+05
Linear	23.08	1	104	<.001	
Quadratic	10.95	1	104	.001	
**Posthoc test**	***t***		***df***	p_{adj}	
+/+ vs. +/-	0.41		104	.684	
+/+ vs. -/-	-4.80		104	<.001	
+/- vs. -/-	-5.37		104	<.001	
**Source (random)**	***N***	***ICC***			
pair:(slice:animal)	107	.14			
slice:animal	56	.33			
animal	22	.06			
Residual		.47			

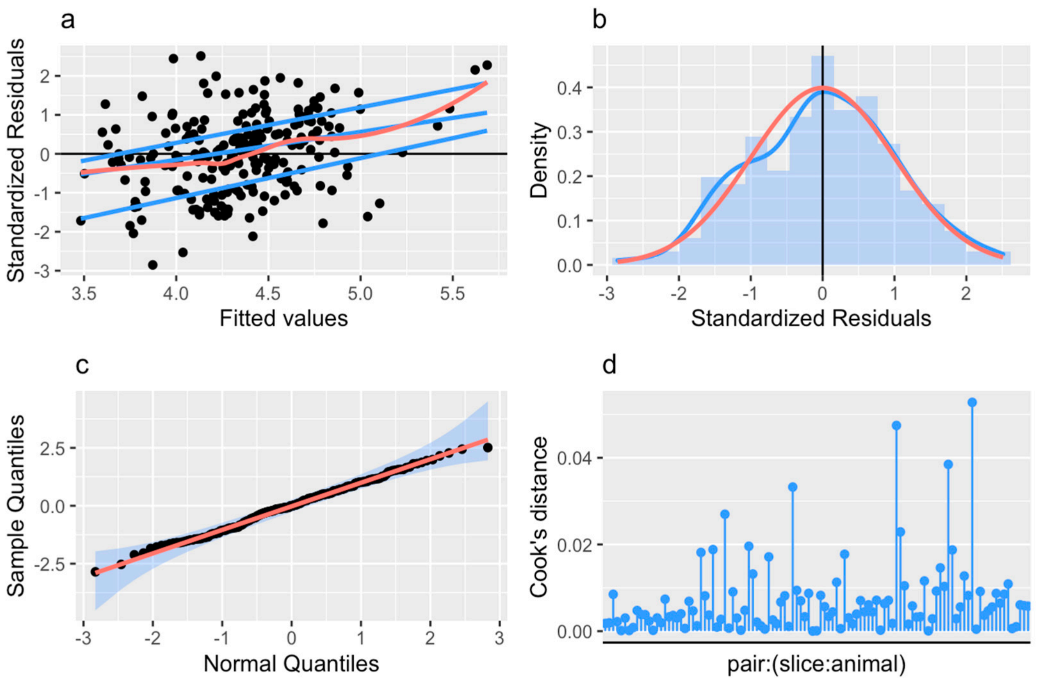

Figure S1.14. NMDA-EPSC decay time constant in neurons expressing GluNB knockout alleles.

Top. Summary tables based on a LMM predicting NMDA-EPSC decay time constant from the transfection of Cre-GFP in CA1 neurons of slices with different genotypes of the floxed *Grin2b* allele. Fixed effects are summarised as an ANOVA table with the interaction term split into polynomial contrasts and followed up with pairwise comparisons by Westfall stepwise posthoc tests. The *p*-value and Bayes factor for the interaction term (genotype:transfection) indicate a highly significant effect of loss-of-function (null) GluN2B alleles on the charge transfer of NMDA-EPSCs. Of the variance not accounted for by the fixed effects, 5%, 28%, 0% and 67% was explained by variability between recording pairs, slices, animals, and residual (unexplained) variance respectively. ***Bottom***. Model residuals appeared to be homoscedastic (**a**), normally distributed (**b**, **c**), without overt outliers (**a, c**) and without influential data points (i.e. Cook's distance < 1.0) (**d**). A breakdown of samples sizes is reported in Table S3.

**Response**					
NMDA-EPSC decay time constant (decay)					
**Formula**					
$\log(\boldsymbol{decay}) \sim genotype * transfection + (1 \vert animal/slice/pair)$					
**Source (Fixed)**	***F***	***df***	***df$_{res}$***	***p***	***BF$_{10}$***
genotype	18.41	2	12.1		
transfection	67.22	1	104		
genotype:transfection	35.84	2	104	<.001	9.43E+11
Linear	66.97	1	104	<.001	
Quadratic	3.59	1	104	.061	
**Posthoc test**	***t***		***df***	***p$_{adj}$***	
+/+ vs. +/-	-2.48		104	.015	
+/+ vs. -/-	-8.18		104	<.001	
+/- vs. -/-	-5.87		104	<.001	
**Source (random)**	***N***	**ICC**			
pair:(slice:animal)	107	.05			
slice:animal	56	.28			
animal	22	.00			
Residual		.67			

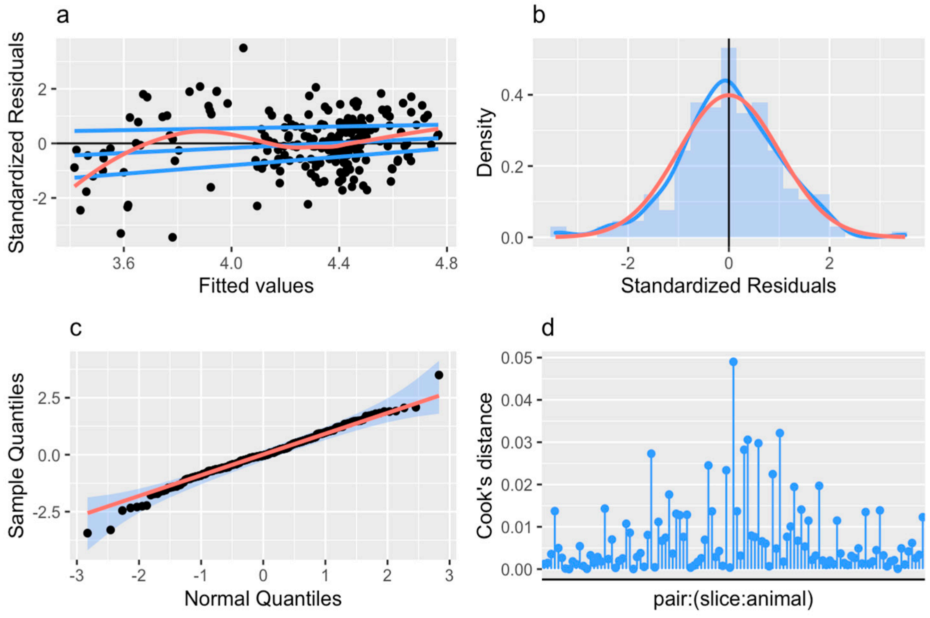

Figure S1.15. NMDA-EPSC risetimes in neurons expressing GluN2B knockout alleles.

Top. Summary tables based on a LMM predicting NMDA-EPSC rise time from the transfection of Cre-GFP in CA1 neurons of slices with different genotypes of the floxed *Grin2b* allele. Fixed effects are summarised as an ANOVA table with the interaction term split into polynomial contrasts and followed up with pairwise comparisons by Westfall stepwise posthoc tests. The *p*-value and Bayes factor for the interaction term (genotype:transfection) indicate a significant effect of loss-of-function (null) GluN2B alleles on the rise time of NMDA-EPSCs. Of the variance not accounted for by the fixed effects, 5%, 55%, 0% and 40% was explained by variability between recording pairs, slices, animals, and residual (unexplained) variance respectively. ***Bottom***. Model residuals appeared to be homoscedastic (**a**), normally distributed (**b**, **c**), without overt outliers (**a, c**) and without influential data points (i.e. Cook's distance < 1.0) (**d**). A breakdown of samples sizes is reported in Table S3.

**Response**					
NMDA-EPSC 20-80% risetime (rise)					
**Formula**					
$\log(\boldsymbol{rise}) \sim genotype * transfection + (1 \vert animal/slice/pair)$					
**Source (Fixed)**	***F***	***df***	$\boldsymbol{df_{res}}$	***p***	$\boldsymbol{BF_{10}}$
genotype	0.52	2	12.13		
transfection	15.67	1	104		
genotype:transfection	6.50	2	104	.002	28.2
Linear	12.72	1	104	<.001	
Quadratic	0.16	1	104	.69	
**Posthoc test**	***t***		***df***	$\boldsymbol{p_{adj}}$	
+/+ vs. +/-	-1.45		104	.15	
+/+ vs. -/-	-3.57		104	.002	
+/- vs. -/-	-2.18		104	.03	
**Source (random)**	***N***	**ICC**			
pair:(slice:animal)	107	.05			
slice:animal	56	.55			
animal	22	.00			
Residual		.40			

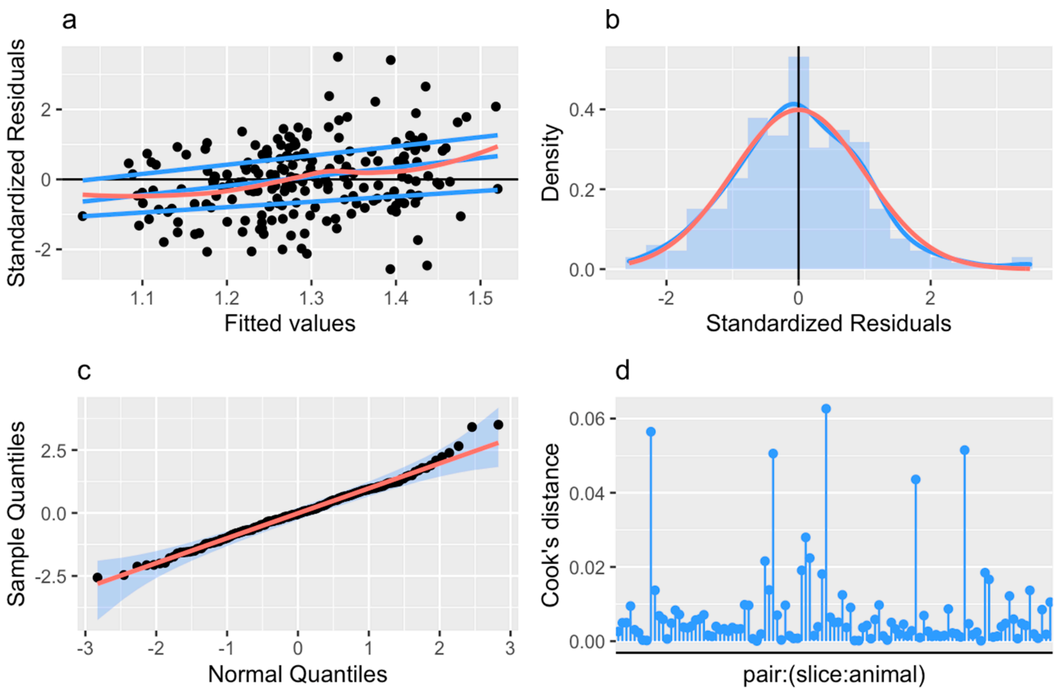

Figure S2: Effective rescue of native mouse GluN2B by human GluN2B

a) The experimental approach used to investigate the effect of missense mutations, by co-expressing Cre-GFP with human *GRIN2B* cDNA by single-cell electroporation around day 7 *in vitro*. The action of Cre recombinase results in the decline of native mouse GluN2B expression in Cre-transfected neurons of organotypic slices from *Grin2b*$^{fl/fl}$ (as well as GluN2A in *Grin2a*$^{fl/fl}$ *b*$^{fl/fl}$) mice slices. Meanwhile, native GluN2B is replaced by the co-expressed human GluN2B.
b) The transfected/untransfected ratios for NMDAR-EPSC peak amplitude and weighted decay time constants for varying amounts of co-transfected human *GRIN2B* cDNA. Arrow indicates the concentration that effectively rescued the peak amplitude and decay time constant of NMDAR-EPSCs. Error bars are 95% CI. The grey arrow indicates the concentration of *GRIN2B* cDNA used to rescue NMDA-EPSCs.
c) Example traces of NMDA-EPSCs measured at +20 mV to illustrate rescue of peak amplitude (left) and peak-scaled to illustrate rescue of the decay kinetics (*right*), in GluN2B KO cells by human GluN2B.

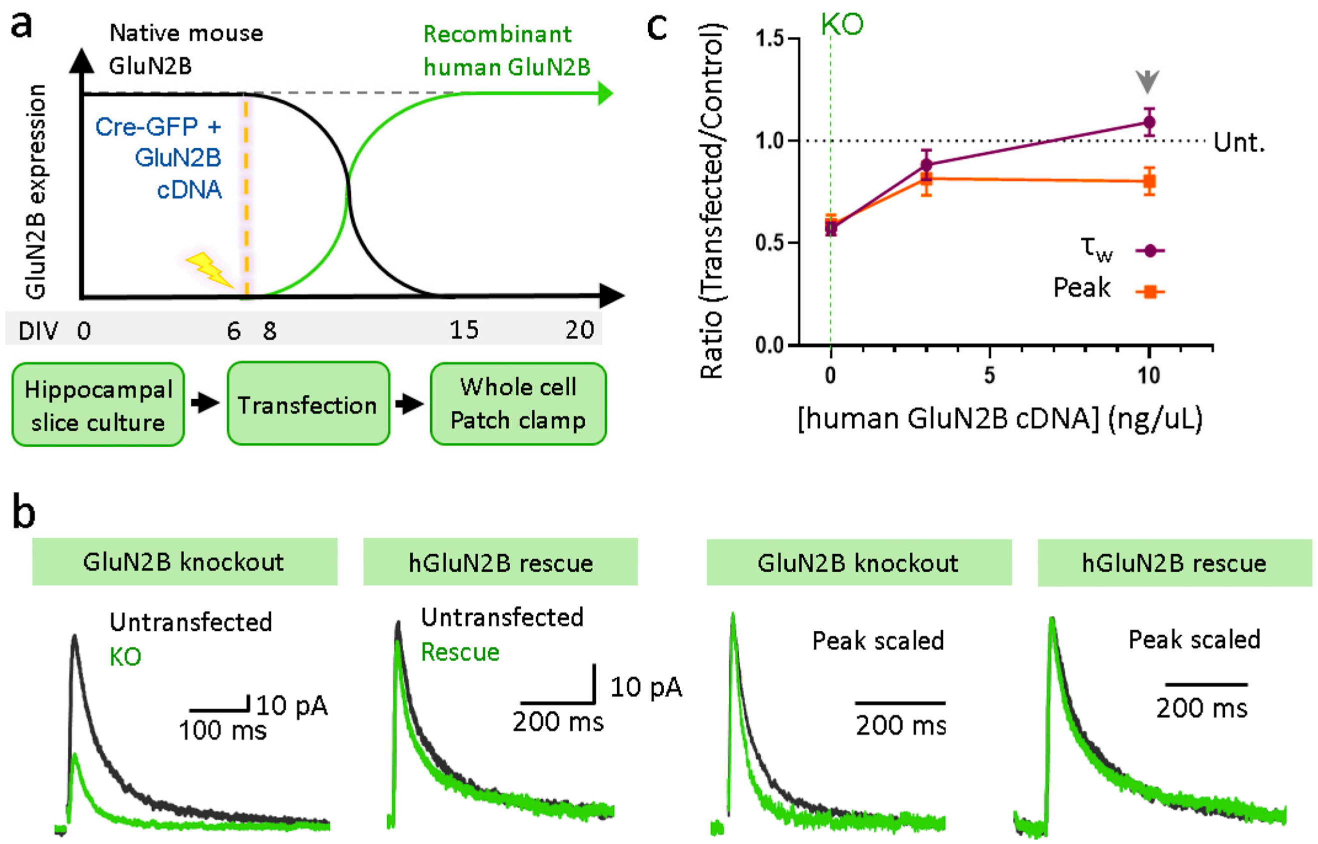

Figure S3: GluN2B mutations are not associated with much effect on AMPAR-EPSCs.

ai) Data points of measurements made in individual neurons. Matched data points, for simultaneously recorded untransfected and transfected neurons, are connected by a line. **aii)** Response ratios (transfected/untransfected) are expressed as a percentage and plotted for each pair of transfected-untransfected neurons. Crossbars in **i)** and **ii)** show the estimated marginal means with 95% confidence intervals backtransformed from the linear mixed models (Figure S1). Hypothesis tests are orthogonal contrasts based on *a priori* clustering of the mutations. Standardized effect sizes (*r*) for comparisons of each mutant with WT for response ratios of peak amplitude in **aii** were +.06, +.09, +.10, and -.10 (*N* = 95).

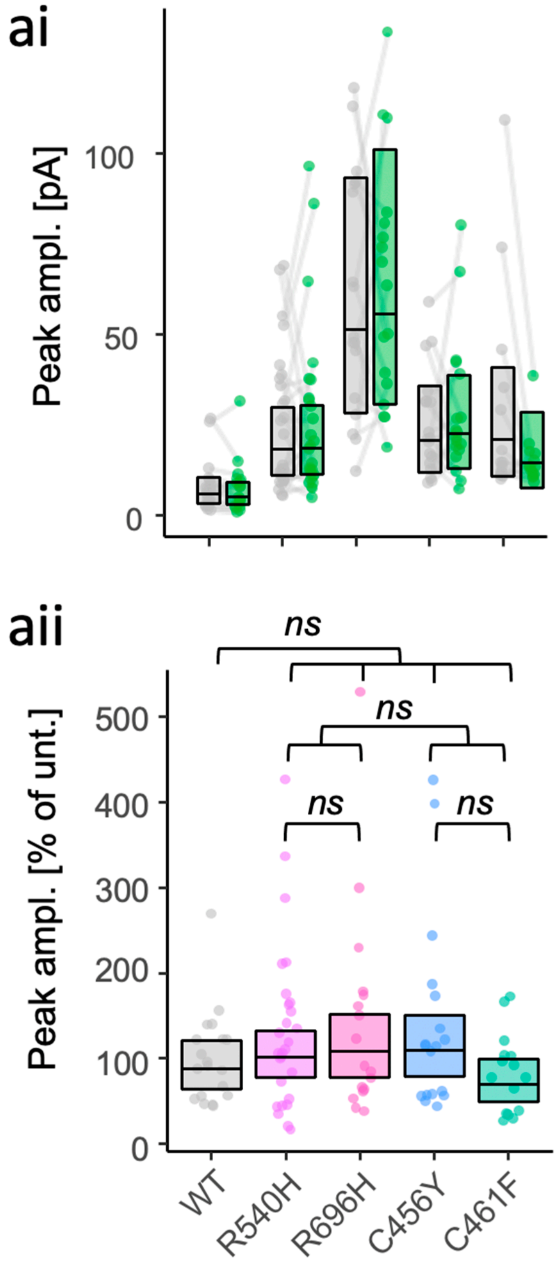

Figure S4: NMDA-EPSC decay is similar after selective inhibition of GluN2A-containing NMDA receptors

ai) NMDA-EPSC decay time constants before, and 13.5 to 17.5 minutes after, the addition of 10 μM TCN-201. Each point represents the weighted decay time constant of the ensemble mean of 40 NMDA-EPSCs. Each pair of points connected by a line correspond to measurements made from individual neurons before and after addition of TCN-201.
aii) Response ratios (after/before) calculated from (ai) are expressed as a percentage and plotted for each pair of before-after recordings. Crossbars show the estimated marginal means with 95% confidence intervals backtransformed from the linear mixed models. Standardised effect sizes (*r*) for each mutant compared to WT for were -.04 and -.10 for mutants R696H and C456Y respectively (*N* = 38).

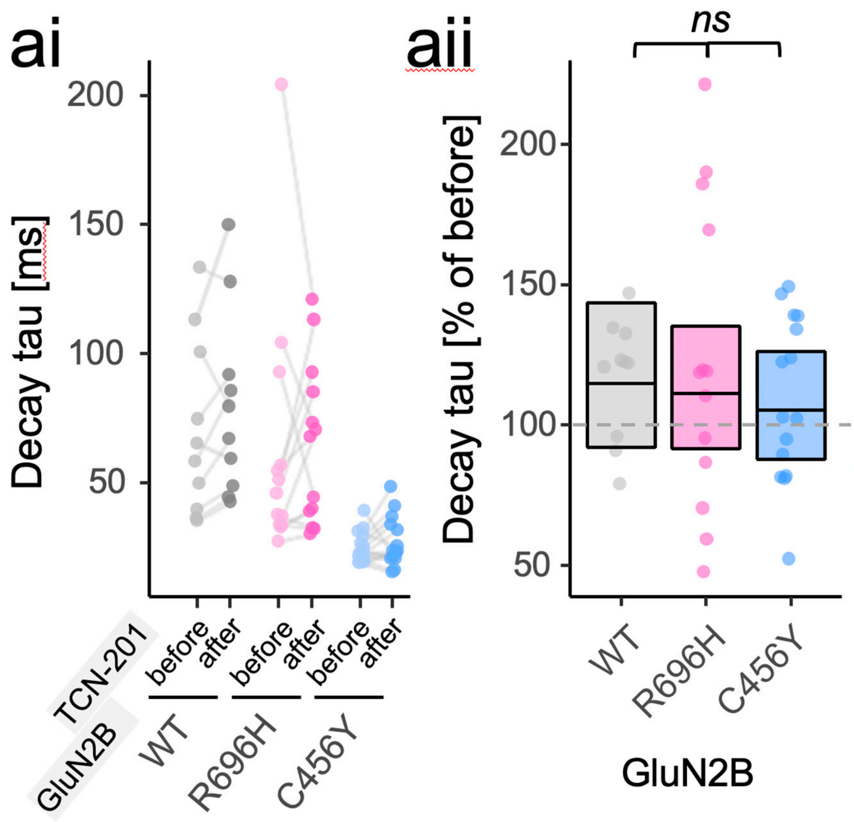

Table S1. Matrix of orthogonal contrasts based on a priori clustering of mutations.

Matrix of orthogonal contrasts used in the analysis of data in Figure 2. Conditional table formatting is used to colour code the mutants involved in each contrast and illustrate their weighting.

**Contrast**	**A**	**B**	**C**	**D**
GRIN2B	**WT *vs* Mutants**	**LoF *vs* GoF**	**LoF**	**GoF**
**WT**	-0.8	0	0	0
**R540H**	0.2	0.5	0	-0.5
**R696H**	0.2	0.5	0	0.5
**C456Y**	0.2	-0.5	-0.5	0
**C461F**	0.2	-0.5	0.5	0

Table S2: Primers used to generate mutant GluN2B constructs from WT pCI-Neo GRIN2B

GRIN2B **MUTATION**	**Forward primer (5' to 3')**	**Reverse primer (5' to 3')**
**R540H**	GTGTCATGGTGTCACACAGCAATGGGACTGT	ACAGTCCCATTGCTGTGTGACACCATGACAC
**R696H**	CAGAGAGAAATATTCACAATAACTATGCAGA	TCTGCATAGTTATTGTGAATATTTCTCTCTG
**C456Y**	GTTACATCAAAAAATACTGCAAGGGGTTCTG	CAGAACCCCTTGCAGTATTTTTTGATGTAAC
**C461F**	GCTGCAAGGGGTTCTTTATTGACATCCTTAA	TTAAGGATGTCAATAAAGAACCCCTTGCAGC
**C436R**	AGGAACACAGTCCCCCGCCAAAAACGCATAG	CTATGCGTTTTTGGCGGGGGACTGTGTTCCT

Table S3. Experiment sample sizes

Sample sizes for experiments detailed in Figures S1.1-1.15 broken down into the number of recording cells (or cell pairs), number of slices, and number of animals. Abbreviations: DKO: GluN2A/B double knockout.

Experiment relating to Figure 1			
Mutation	pairs	slices	animals
None (DKO)	19	11	3
WT	19	13	3
R540H	12	9	2
R696H	16	10	3
C456Y	24	14	3

Experiment relating to Figure 2 and S3			
Mutation	pairs	slices	animals
WT	19	12	4
R540H	27	18	6
R696H	17	11	4
C456Y	18	12	5
C461F	15	9	3

Experiment relating to Figure 3			
Genotype	cells	slices	animals
WT	10	7	1
R696H	13	10	2
C456Y	15	9	2

Experiment relating to Figure 5			
Mutation	pairs	slices	animals
C436R (N2B)	16	9	3
C436R (N2A)	17	12	6

Experiment relating to Figure 6			
Genotype	pairs	slices	animals
WT	33	19	4
HET	37	18	11
HOM	37	19	7

Table S4. Functional properties of NMDA receptor subtypes in HEK293T cells

Responses from NMDA receptor subtypes were measured using fast-application whole-cell patch-clamp recordings and activated by brief 5 ms application of 1 mM glutamate in the continuous presence of 100 µM glycine. Rise times are for 10–90% of the peak response amplitude, deactivation time constants were determined using two-exponential fits to obtain τ_{fast}, τ_{slow}, and % fast is the fitted percentage of the fast component. [a] indicates that only a single-exponential was fitted to the deactivation time course. Decay time is the weighted time constant ($\tau_{weighted}$) (see Materials and Methods). Data are mean ± *SEM*, and *N* is the number of cells used to generate the data. Statistical tests on the log_{10}-transformed weighted decay time constants are described in the legend of Fig. 4.

Receptor	Rise time (ms)	τ_{fast} (ms)	τ_{slow} (ms)	% fast	Decay time $\tau_{weighted}$ (ms)	*N*
GluN1/2B^{WT}	11.3 ± 0.8	238 ± 12	884 ± 68	65 ± 4	443 ± 15	14
GluN1/2B^{R540H}	14.7 ± 0.9	456 ± 47	1421 ± 288	62 ± 5	833 ± 60	8
GluN1/2B^{R696H}	4.6 ± 0.9	235 ± 31	1281 ± 264	55 ± 7	615 ± 41	7
GluN1/2A/2B^{WT}	5.6 ± 0.3	41 ± 6	397 ± 197	90 ± 3	56 ± 6	7
GluN1/2A/2B^{R540H}	7.0 ± 0.8	37 ± 9	331 ± 102	83 ± 7	59 ± 9	7
GluN1/2A/2B^{R696H}	6.3 ± 0.3	44 ± 5	244 ± 36	80 ± 8	74 ± 9	8
GluN1/2A	5.8 ± 0.8	45 ± 4	-	100[a]	45 ± 4	9

Table S5. Effects of TCN-201 on responses from NMDA receptors in HEK293T cells

Responses from NMDA receptor subtypes were measured using fast-application whole-cell patch-clamp recordings. Responses in the absence and presence of 5 μM TCN-201 were activated by brief 5 ms application of 1 mM glutamate in the continuous presence of 3 μM glycine. Deactivation time constants were determined using two-exponential fits, but the decay time ($\tau_{weighted}$) is listed (see Materials and Methods). n.d. indicates that reliable measurements of the decay time could not be made because sufficient currents could not be detected. Data are mean ± *SEM*, and *N* is the number of cells used to generate the data. The decay data were analysed by two-way (mixed model) ANOVA (Type III) testing the effects of TCN-201 on measurements of $\log_{10}$-transformed weighted decay time constants across the different receptors tested. Repeated measures were modelled for before and after application of TCN-201. We found no significant main effect of TCN-201 on the decays ($F(1,26) = 0.032$, $p = .86$) but did find a highly significant difference in the decays across the different receptors tested ($F(5,26) = 111.2$, $p = <.001$), consistent with our results in Fig. 4. Interpretation of the main effects was simplified by there being no statistically significant TCN-201 x receptor interaction ($F(5,26) = 0.66$, $p = .65$). Refer to the legend of Fig. 4 for the statistics on the % inhibition of the peak amplitude.

Receptor	Decay time $\tau_{weighted}$ (ms)		% inhibition	*N*
	Before TCN-201	After TCN-201		
GluN1/2B^{WT}	365 ± 12	355 ± 27	4 ± 4	5
GluN1/2B^{R540H}	675 ± 67	613 ± 105	19 ± 5	5
GluN1/2B^{R696H}	490 ± 24	462 ± 24	4 ± 6	5
GluN1/2A/2B^{WT}	50 ± 2	60 ± 2	70 ± 6	6
GluN1/2A/2B^{R540H}	58 ± 12	68 ± 11	82 ± 6	5
GluN1/2A/2B^{R696H}	65 ± 8	67 ± 10	70 ± 6	6
GluN1/2A	40 ± 3	n.d.	95 ± 2	4
